# SERS Activity in Gold Particles Obtained via a Modified Seeded-Growth Method

**DOI:** 10.1021/acsomega.6c04232

**Published:** 2026-06-09

**Authors:** Jathziri Avalos-Grajales, Mario Alejandro Millán-Franco, Lucia Ortega-Cabello, Edgar Eduardo Mosquera-Vargas, José Reyes-Gasga

**Affiliations:** † Instituto de Física, Universidad Nacional Autónoma de México, 04510 Ciudad de México, México; ‡ Departamento de Sistemas Biológicos, Universidad Autónoma Metropolitana, 04960 Ciudad de México, México; § Grupo de Transiciones de Fase y Materiales Funcionales (GTFMF), Departamento de Física, 28006 Universidad Del Valle, Santiago de Cali 760032, Colombia; ∥ Centro de Excelencia en Nuevos Materiales (CENM), Universidad Del Valle, Santiago de Cali 760032, Colombia

## Abstract

Precise control over nanoparticle morphology and spatial arrangement is fundamental for optimizing plasmonic performance. This study investigates the role of the HAuCl_4_/NaBH_4_ molar ratio in governing the formation kinetics, structure and Surface-Enhanced Raman Scattering (SERS) response of gold nanoparticles (AuNPs) synthesized via seed-mediated approach in which triangular gold nanoparticles (T-AuNPs) account for an increased fraction. Real-time ultraviolet–visible (UV–Vis) spectroscopy reveals a direct correlation between absorbance evolution and precursor consumption, consistent with the Beer–Lambert law, and identifies distinct kinetic regimes controlled by reductant concentration. At low NaBH4 content (1:12), nucleation proceeds under diffusion-limited conditions, yielding small and relatively uniform nanoparticles. Intermediate ratios (1:6) promote chemically controlled growth, while equimolar (1:1) conditions induce delayed nucleation followed by rapid growth, resulting in larger triangular nanoplates with broader size distributions. Electron microscopy (scanning electron microscope (SEM) and HR-transmission electron microscopy (TEM)) confirms that these structures enhance plasmonic coupling at sharp edges and interparticle junctions, generating intense electromagnetic hot spots. Consequently, the equimolar (1:1) system exhibits up to a 9-fold increase in SERS intensity compared to more dispersed nanoparticle assemblies. These findings demonstrate that kinetic regulation provides an effective bottom-up strategy to direct anisotropic growth and tune plasmonic properties without postsynthetic modification, enabling the development of highly sensitive SERS substrates for molecular detection and plasmonic sensing.

## Graphical abstract

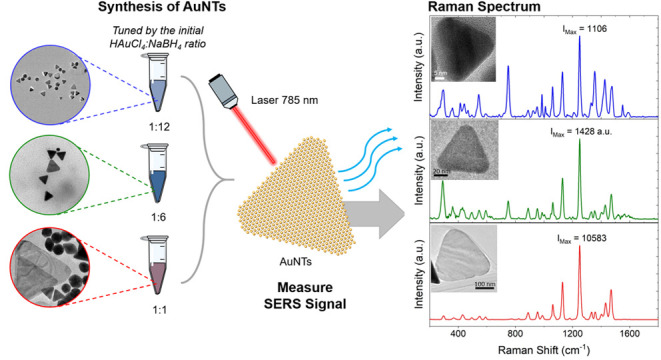

## 1 Introduction

One of the primary goals in materials science is the controlled synthesis of nanostructures with defined shapes and sizes, as morphology governs their optical, electronic, catalytic, and mechanical properties.^1^ Gold nanoparticles (AuNPs) are particularly attractive due to their physicochemical stability, tunable localized surface plasmon resonance (LSPR), and diverse applications in photonics,^2,3^ catalysis,^4,5^ sensing,^6^ and nanomedicine.^7–9^

Over the last two decades, numerous anisotropic Au nanostructures -including nanocubes,^10^ nanorods,^11,12^ octahedra,^13,14^ and decahedra^15^ as well as nanospheres,^16^ have been synthesized through wet-chemical methods, enabling precise morphological and plasmonic control. Among these, triangular gold nanoparticles (T-AuNPs), such as nanotriangles (AuNTs) or nanoplates (AuNPs), possess extended {111} facets and sharp vertices that support both in-plane and out-of-plane plasmon modes, producing strong electromagnetic field localization.^17^

These optical features make them highly suitable for surface-enhanced Raman scattering (SERS), biosensing, and imaging applications.^18,19^ The pronounced anisotropy of triangular particles induces strong charge localization at the edges and corners, generating electromagnetic “hot spots” that enhance Raman signals by several orders of magnitude, making them effective SERS substrates.^20–22^ Furthermore, their tunable geometry enables diverse applications in imaging,^23^ catalysis,^24^ and chemical or biological sensing.^25,26^

Beyond this general behavior, the intensity of electromagnetic enhancement in SERS is strongly governed by the local geometry of plasmonic nanostructures. Sharp features such as edges, corners, and vertices promote charge accumulation via the lightning-rod effect, giving rise to highly localized near fields. In contrast, nanoparticles with rounded morphologies, such as spheres or low-curvature structures, exhibit a more homogeneous surface charge distribution and therefore generate weaker local field enhancements.^27,28^

Consequently, controlling the formation of high-curvature features, for example, the acute angles of triangular gold nanoparticles or the sharp tips of nanostars, is critical for maximizing plasmonic performance and SERS activity.^29^ This strong geometric dependence underscores the importance of understanding how reaction kinetics govern not only particle size but also shape anisotropy, as these structural parameters directly determine the efficiency of electromagnetic enhancement mechanisms.

However, obtaining high-yield, monodisperse AuNPs remains challenging because anisotropic growth is not thermodynamically favored in face-centered cubic (FCC) structures; instead, it requires precise kinetic control and facet-selective stabilization.^30,31^ Modified seed-mediated growth methods based on the work of Scarabelli and Liz-Marzán,^32^ which utilize preformed spherical nuclei (≤3 nm), enable anisotropic growth under controlled reduction and capping conditions. However, these methods typically involve multiple steps and are sensitive to seed again, reagent purity, and injection rate-factors that frequently limit both yield and scalability.

Seedless growth via oxidative etching has also been proposed as an alternative. Chen et al.^33^ demonstrated that iodide ions (I^–^) selectively stabilize {111} facets while forming triiodide (I_3_^–^) species. These species etch unstable nuclei, thereby favoring planar-twinned seeds and yielding >90% triangular particles. These results highlight the critical role of chemical etching and facet-selective stabilization in dictating nanoparticle symmetry.

A mechanistic understanding of reaction kinetics is therefore necessary to correlate synthesis parameters with morphology and optical response.^13,29,34^ Precursor concentration, reducing agent concentration, and reaction time control the nucleation–growth transition, ultimately determining the shape and size of the resulting T-AuNPs.^35–37^ UV–vis spectroscopy enables real-time monitoring of reaction progress and kinetic modeling via pseudo-first- or second-order rate laws. This approach provides critical insights into rate-limiting steps and growth constants, which are essential for ensuring reproducibility and scalability.^38,39^

This manuscript reports a seeded-growth strategy to control the morphology of gold nanoparticles by tuning the HAuCl_4_: NaBH_4_ molar ratio, and correlates the reaction kinetics with particle shape and SERS performance. By combined UV–vis kinetic analysis with electron microscopy (SEM and TEM) characterization, we establish a direct correlation between reaction kinetics, morphology, and plasmonic response. This approach enables the room-temperature preparation of highly SERS-active triangular particles and provides practical guidelines for the scalable fabrication of plasmonic nanostructures. The results suggest that adjusting the precursor ratio can modulate nucleation–growth regimes and consequently influence plasmonic properties.

## 2 Experimental Section

Figure 1 shows the schematic representation of the seed-mediated synthesis for the gold nanoparticles. The diagram illustrates the general reaction pathway; exact reagent concentrations, volumes, and synthesis conditions (1:1, 1:6, and 1:12). Unlike conventional protocols that require purification or complex reagents, this method minimizes processing steps while maintaining high structural quality and reproducibility.

**Figure 1:**
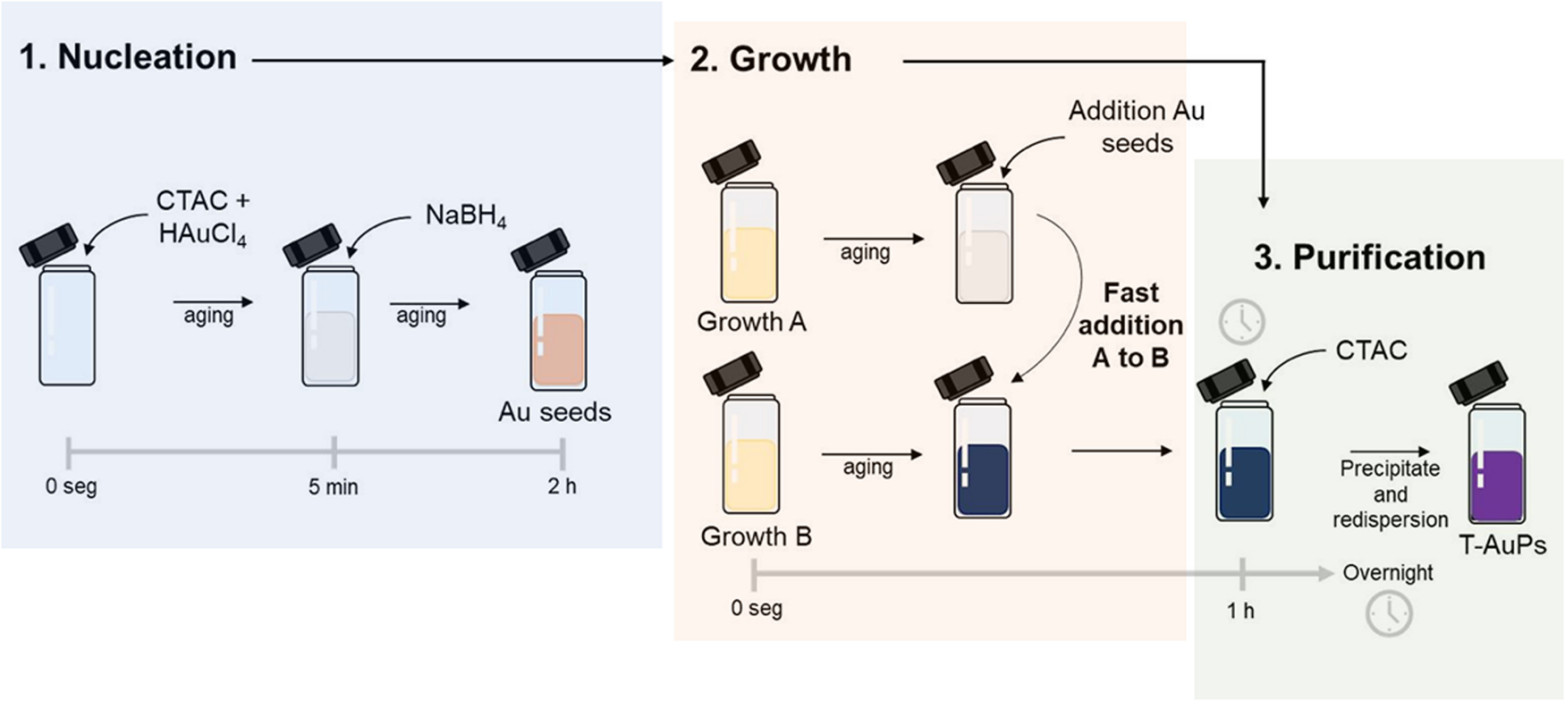
Schematic representation of the seed-mediated synthesis for the gold nanoparticles. The reaction pathway; exact reagent concentrations, volumes, and synthesis conditions (1:1, 1:6, and 1:12) are detailed in text.

### 2.1 Chemicals

Hydrogen tetrachloroaurate (III) trihydrate (HAuCl_4_·3H_2_O), l-Ascorbic acid (AA), Sodium iodide (NaI), and Sodium borohydride (NaBH_4_) were purchased from Sigma-Aldrich. Hexadecyltrimethylammonium chloride (CTAC, 25 wt % in water) was obtained from Econoclear. All chemicals were of analytical grade and used as received without further purification. Deionized water was used for all the synthesis procedures and sample preparations.

### 2.2 Synthesis

CTAC was employed as a capping and stabilizing agent to promote anisotropic growth of T-AuPs. The synthesis consisted of two stages (Figure 1): (i) seed preparation and (ii) particles growth. First, a seed solution was prepared by mixing 300 μL of freshly prepared 10 mM NaBH_4_ with 4.7 mL of 100 mM CTAC and 25 μL of 50 mM HAuCl_4_. The mixture was vigorously stirred for 2 h at room temperature (RT) to ensure complete formation and stabilization of the initial nuclei. Additional HAuCl_4_/NaBH_4_ molar ratios (1:1 and 1:6) were also prepared to obtain seeds of varying sizes.

In the growth step, two separate solutions were prepared. Solution A contained 1.6 mL of 100 mM CTAC, 8 mL of distilled water, 40 μL of 50 mM HAuCl_4_, and 15 μL of 10 mM NaI, followed by the addition of 40 μL of 100 mM AA. Solution B consisted of 20 mL of 100 mM CTAC stock solution, 20 mL of deionized water, 500 μL of 50 mM HAuCl_4_, and 300 μL of 10 mM NaI, followed by the addition of 400 μL of 100 mM AA. Finally, 100 μL of the previously diluted seed solution was added to Solution A to initiate growth. Subsequently, 3.2 mL of Solution A was rapidly transferred into Solution B under stirring to facilitate further particles growth and the formation of triangular structures.

### 2.3 Purification

To the colloidal suspension, 630 μL of the 25 wt % CTAC solution was added, and the mixture allowed to stand for 18 h. The supernatant was subsequently removed, leaving a dark precipitate, which was then redispersed in 5 mL of distilled water.

### 2.4 Characterization

UV–vis spectra were recorded between 200 and 900 nm using a Thermo Scientific Evolution 200 spectrophotometer with a 1 cm path-length cuvette and a NanoDrop 2000c (2 μL, sample volume).to monitor the evolution of the LSPR. Morphology and size distribution were analyzed via scanning electron microscopy (SEM, JEOL JSM-7800) and transmission electron microscopy (TEM, JEOL 2010 FEG). Electron microscopy samples were prepared by depositing 10 μL of the colloidal suspension onto SEM stubs or carbon-coated copper grids and drying under ambient conditions. Prior to deposition, excess surfactant was removed through three centrifugation-redispersion cycles.

SERS measurements were performed using a JASCO NRS-4500 Raman spectrometer equipped with a 784.98 nm laser (1.58 eV). Spectra were collected with a laser power of 2.7 mW, an exposure time of 60 s, and five accumulations. Raman spectra were recorded in the 200–1800 cm^–1^ range to evaluate plasmonic enhancement.

## 3 Results and Discussion

### 3.1 Reaction Kinetics and Growth Regimes

The formation of gold nanoparticles can be described as a two-stage process consisting of nucleation and growth, where each stage is governed by distinct kinetic regimes. Nucleation involves the initial reduction of Au^3+^ species and the formation of stable nuclei, while the growth stage is dominated by surface-mediated reduction and particle enlargement.

This process was monitored in situ using time-resolved UV–vis spectroscopy to track precursor consumption and growth evolution across the different molar ratios. Absorbance changes were correlated with the HAuCl_4_, concentration via the Beer–Lambert Law, enabling a quantitative analysis of the reaction progress. All experiments were performed in triplicate, and the presented values represent the calculated averages.

Kinetic analysis was conducted for each HAuCl_4_/NaBH_4_ molar ratio using apparent reaction-order models. While the initial HAuCl_4_ concentration was varied, the CTAC, NaI, and AA concentrations were kept constant. Linear regressions were applied to both pseudo-first-order models

$$\ln\left(A_{t}\right)=-k^{\prime }t+\ln\left(A_{0}\right)$$

and pseudo-second-order models

$$\left(\frac{1}{A_{t}}=k^{\prime }t+\frac{1}{A_{0}}\right)$$

where *A*_*t*_ is the absorbance at time *t* and *A*_0_ is the initial absorbance.

Kinetic parameters were extracted from linear fits of the absorbance–time profiles at characteristic temporal regions. The initial stage was associated with precursor depletion and intermediate formation (nucleation regime), whereas the later stage corresponded to plasmon development (growth regime). Although partial temporal overlaps may occur, each stage was treated as a quasi-independent regime based on dominant spectral contributions. The apparent rate constant (*k* ^′^) and coefficient of determination (*R* ^2^) were obtained from the linear fits, and the best-fitting kinetic model was selected based on the highest *R* ^2^ value (Table 1). Additional details and fitting data set are provided in Table S1 (Supporting Information), which is a comprehensive overview of the entire kinetic study.

**Table 1:** Kinetic Parameters Derived from the Linear Fits of the Pseudo-First-Order and Pseudo-Second-Order Models for Different HAuCl_4_/NaBH_4_ Molar Ratios at the Corresponding Stages of the Synthesis

moral ratio (HAuCl_4_/NaBH_4_)	*R* ^2^	*k*′	stage of the synthesis	evaluated model
1:12	0.9074	0.0039 ± 4.73 × 10^–4^ mol s^–1^	nucleation	pseudo-second order
	0.9002	0.0028 ± 3.66 × 10^–4^ s^–1^	growth	pseudo-first order
1:6	0.9176	0.002 ± 2.54 × 10^–4^ s^–1^	nucleation	pseudo-first order
	0.9087	0.0027 ± 3.29 × 10^–4^ s^–1^	growth	pseudo-first order
1:1	0.9124	0.0001 ± 0.16 × 10^–4^ mol s^–1^	nucleation	pseudo-second order
	0.9062	0.038 ± 0.0055 s^–1^	growth	pseudo-first order

To assign the spectral features used for kinetic tracking, three reference reaction environments were analyzed. For HAuCl_4_ in distilled water, two absorption peaks at 220 and 294 nm were observed (Figure S1), corresponding to the characteristic electronic transitions of the Au^3+^ precursor.^40,41^ In the presence of CTAC, the spectrum shifted, displaying bands at 220 and 320 nm (Figure S2).

The redshift to 320 nm indicates the formation of the [AuCl_4_]^−^–CTA^+^ ion pair, confirming the interaction between the precursor and the micellar surfactant while defining the spectral signature of the intermediate complex.^42,43^ Upon the addition of NaI and AA, a single band centered at 550 nm appeared (Figure S3),^44,45^ corresponding to the LSPR. This peak indicates the reduction of Au^3+^ to metallic Au^0^ and subsequent nanoparticle growth.^46–48^ Calibration curves were constructed at these selected wavelengths to convert absorbance into relative concentration, allowing for the extraction of kinetic parameters.

The temporal spectral evolution of the reaction for the various HAuCl_4_/NaBH_4_ molar ratios is shown in Figure 2. In the 1:12 synthesis during the nucleation stage (Figure 2a), the band associated with the gold precursor gradually decreases, indicating its consumption; however, no detectable intermediate signal is observed. During the growth stage (Figure 2b), a continuous increase in the plasmonic band is observed, which corresponds to the formation of the nanoparticles.

**Figure 2:**
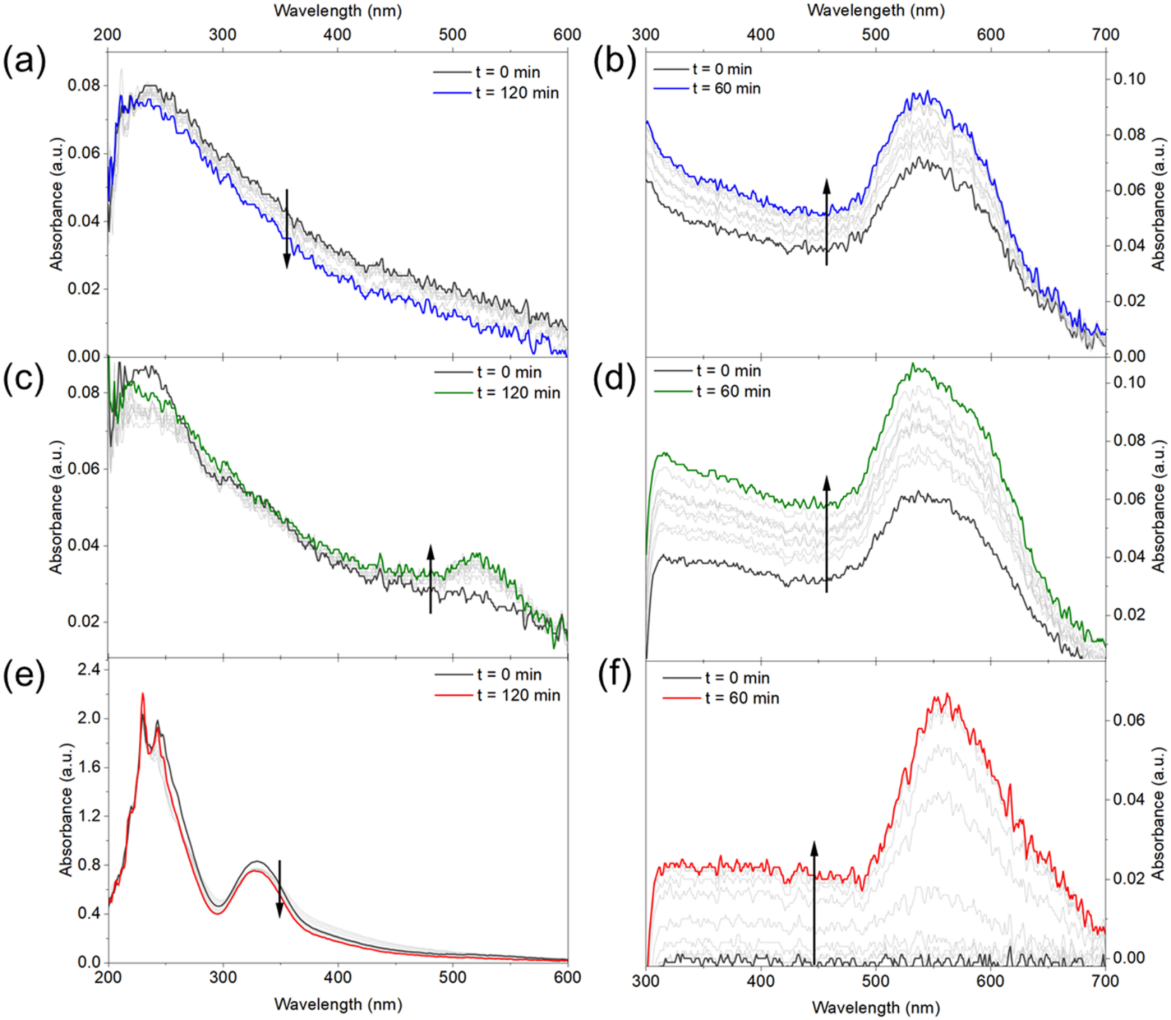
Time-resolved UV–vis spectra illustrating the temporal evolution of nucleation and growth for AuPs at HAuCl_4_/NaBH_4_ molar ratios of 1:12 (a, b), 1:6 (c, d), and 1:1 (e, f). The black arrows denote the direction of spectral shifts over the course of the reaction.

For the 1:6 synthesis, the early reaction period exhibits simultaneous precursor consumption and the appearance of an intermediate spectral contribution (Figure 2c), indicating the localized formation of the precursor-surfactant complex. During the subsequent growth stage (Figure 2d), the plasmonic resonance develops and is slightly red-shifted relative to the 1:12 system, which is consistent with the formation of larger particles.

In the 1:1 synthesis (Figure 2e), two spectral contributions corresponding to the precursor and the intermediate complex are initially observed, both of which decrease over time, indicating the progressive reduction of Au^3+^. During the growth stage (Figure 2f), a well-defined plasmonic band emerges and increases in intensity as metallic nanoparticles nucleate and grow.

The apparent reaction order depends on the HAuCl_4_/NaBH_4_ molar ratio. In the 1:12 system, where NaBH_4_ is in large excess, the reaction initiates rapidly and becomes diffusion-limited (small spheres), as indicated by the nucleation rate constant (*k*′= 3.9 × 10^–4^ mol · s^–1^, *R* ^2^ = 0.9074). This rapid supersaturation produces a high density of Au^0^ nuclei, effectively distributing the available precursor among numerous growth sites. During the growth stage (*k*′ = 2.8 × 10^–3^ s^–1^, *R* ^2^ = 0.9002), reduction occurs primarily at the particle surfaces, yielding small, relatively homogeneous particles.

In the 1:6 system, nucleation (*k*′ = 2.0 × 10^–3^ s^–1^, *R* ^2^ = 0.9176) and growth (*k′* = 2.7 × 10^–3^ s^–1^, *R* ^2^ = 0.9087) occur rapidly and in a balanced manner (triangles), representing the most kinetically favorable regime. The intermediate NaBH_4_ concentration sustains continuous electron transfer without uncontrolled supersaturation, resulting in a chemically controlled regime governed by intrinsic redox kinetics. The pseudo–first-order behavior observed in both stages indicates a single dominant pathway for Au^3+^ reduction. This equilibrium between the reduction rate and precursor availability produces a moderate density of nuclei and promotes uniform anisotropic growth, yielding well-defined triangular particles.

The 1:1 system exhibits the most complex kinetic behavior. Despite the higher concentration of NaBH_4_, the nucleation stage is the slowest among all ratios (*k*′ = 1.0 × 10^–4^ mols^–1^, *R* ^2^ = 0.9124), which can be attributed to the formation of [AuCl_4_]^−^–CTA^+^ ion pairs that stabilize Au^3+^ species and hinder their direct reduction. This stabilization increases the apparent activation barrier for electron transfer, delaying the onset of nucleation. Once these complexes are disrupted, the reaction rate increases dramatically during the growth stage (*k*′ = 3.8 × 10^–2^ s^–1^, *R* ^2^ = 0.9062), indicating a transition to a kinetically complex regime characterized by delayed nucleation followed by accelerated growth. This abrupt increase in reduction rate leads to high supersaturation conditions, favoring rapid deposition of Au^0^ onto a limited number of nuclei and promoting particle growth and interparticle interactions, consistent with the broad and red-shifted LSPR band observed.

Although the kinetic behavior of the 1:1 system was initially described as a burst-growth regime, a more detailed analysis suggests a more complex mechanism. The delayed decrease of the precursor-related absorption bands, followed by a rapid increase in the plasmonic signal, indicates that nucleation is initially hindered, likely due to the stabilization of [AuCl_4_]^−^–CTA^+^ complexes. Once these intermediates are destabilized, the reduction process accelerates, leading to rapid particle growth. However, the broad and red-shifted LSPR band, together with the morphological features observed by SEM and TEM (commented in Figures 4 and 5), suggests that additional processes such as secondary nucleation and particle coalescence may also contribute to the final structure. Interparticle interactions and partial aggregation can enhance plasmonic coupling, resulting in spectral broadening and increased SERS activity. Therefore, the 1:1 system is better described as exhibiting delayed nucleation followed by accelerated growth in a kinetically complex regime, rather than a purely burst-growth process. This interpretation provides a more comprehensive understanding of the interplay between reaction kinetics, particle morphology, and plasmonic response.

### 3.2 Plasmonic Response of the Obtained Gold Particles

Given that the HAuCl_4_/NaBH_4_ molar ratio controls the nucleation and growth regimes, the optical response of the resulting nanoparticles was analyzed to evaluate the effects on their plasmonic properties. UV–vis absorption spectra (Figure 3) showed systematic variations in the position and width of the LSPR band, indicating differences in particle size distribution and morphology.^49,50^ A blueshift and band broadening were observed for the smaller particles,^51,52^ suggesting a heterogeneous population of AuNPs within the colloidal system. Usually, smaller spherical particles exhibit a blueshift compared to larger ones. In comparing triangles to spheres, the shift is often more related to the aspect ratio and sharpness of the tips than just the diameter.

**Figure 3:**
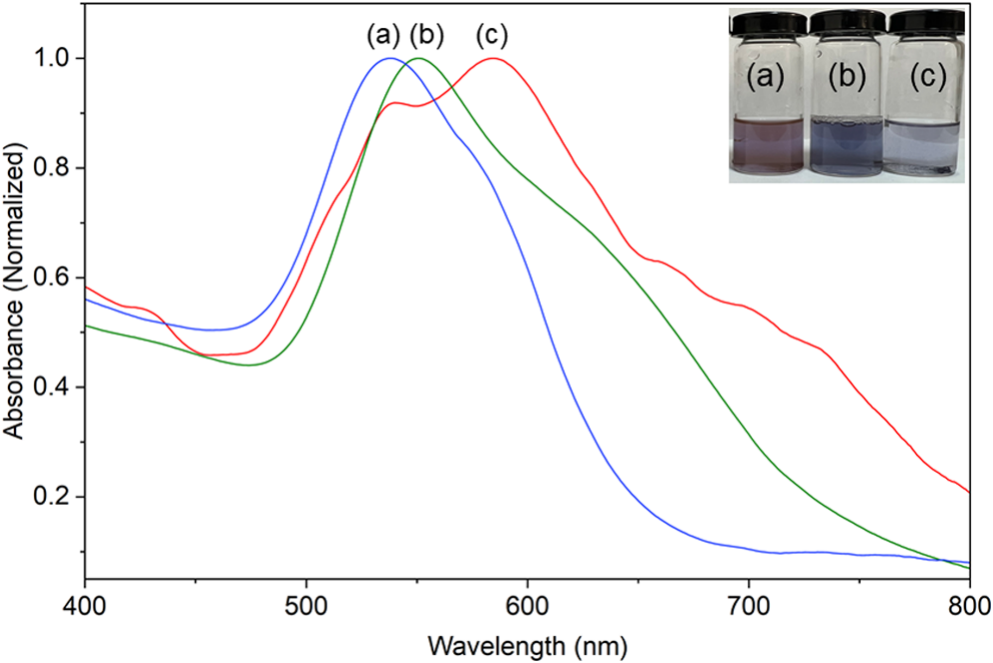
UV–vis spectra of the as-synthesized AuPs at various HAuCl_4_/NaBH_4_ molar ratios: (a) 1:12, (b) 1:6, and (c) 1:1.

For the 1:12 ratio (Figure 3a), the LSPR band is narrow and blue-shifted, red-shifted with smaller and more uniform nanostructures. At the 1:6 ratio (Figure 3b), a similar spectral position is observed; however, a shoulder near the primary resonance suggests moderate size heterogeneity. The spectrum for the 1:1 ratio (Figure 3c) exhibits two distinct plasmon peaks, consistent with a bimodal size distribution and the coexistence of multiple morphologies. This dual plasmonic response is typically associated with overlapping growth regimes and incomplete shape control.^53^

### 3.3 SEM Morphological Analysis

To verify the structural implications inferred from the plasmonic response, the morphology of the gold particles synthesized at different HAuCl_4_/NaBH_4_ molar ratios was examined using SEM. These images allow for the direct visualization of particle shape and size distribution, providing physical confirmation of the variations suggested by the optical spectra.

Figure 4 shows representative backscattered electron (BSE) SEM images of the various HAuCl_4_/NaBH_4_ molar ratios. In Figure 4a,c, multiple shapes of the nanoparticles can be observed, in which T-AuPs account for a small fraction. For ratio 1:12 (Figure 4a), the image shows multiple shapes of the nanoparticles -spherical and decahedral- and a very small fraction of T-AuPs. This suggests the coexistence of multiple nucleation and growth mechanisms, resulting in particles with varied morphologies but similar dimensions. Statistical analysis performed on more than 200 triangular particles (Figure 4b) yielded a Gaussian distribution with an average side length -measured from the vertex to the base- of 31.3 ± 5.06 nm.

**Figure 4:**
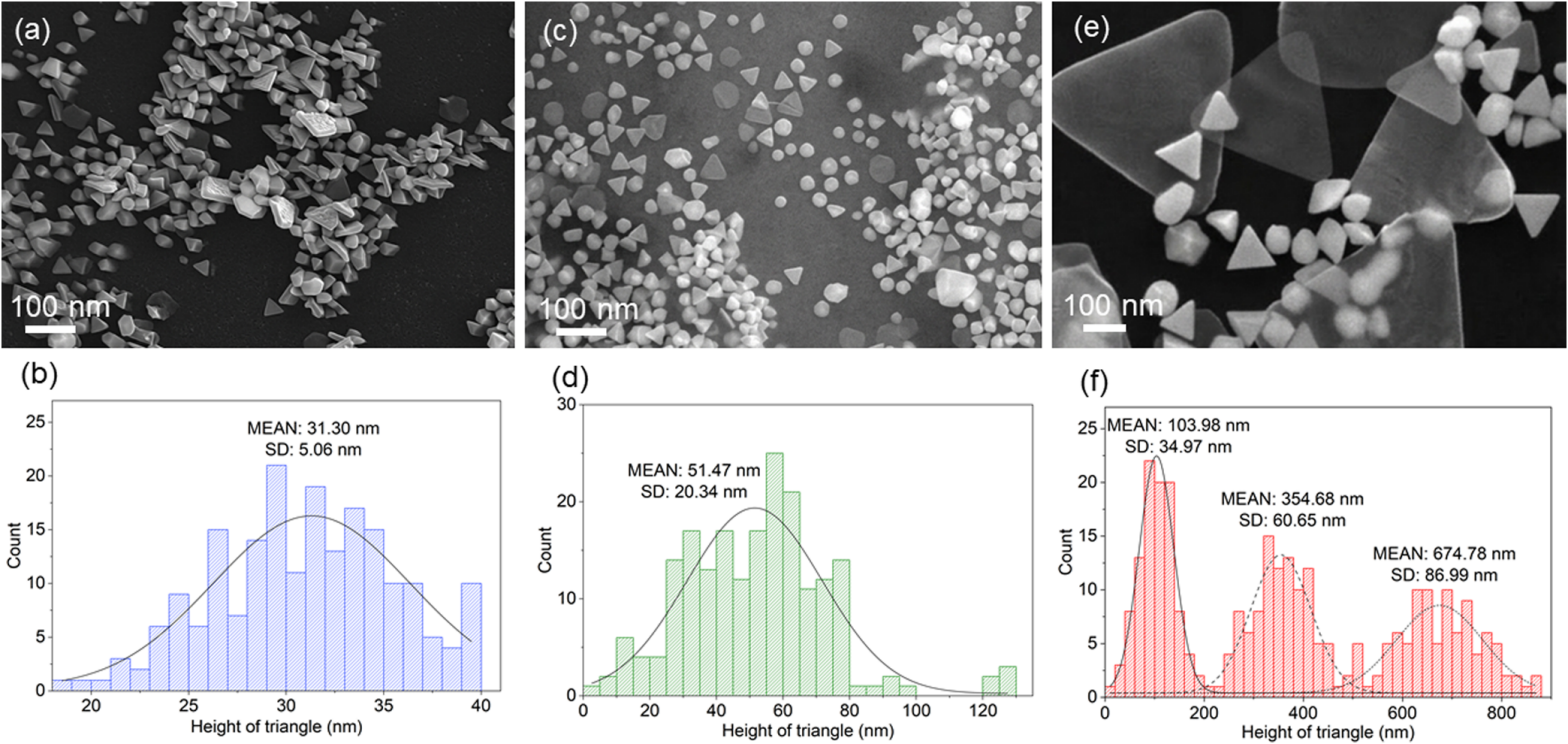
BSE-SEM images and particle size histograms of the T-AuPs synthesized at HAuCl_4_/NaBH_4_ molar ratios of (a, b) 1:12, (c, d) 1:6, and (e, f) 1:1. Histograms for the 1:12 and 1:6 ratios were derived from 200 particles each, while the 1:1 was based on 300 particles. In (a, c), multiple shapes of the nanoparticles can be observed, in which T-AuPs account for a small fraction.

For the 1:6 molar ratio (Figure 4c), the particles exhibit larger, less-defined structures comprising triangular, polygonal, and irregularly shaped particles. The corresponding histogram for the triangular particles (Figure 4d) shows a broader size distribution that follows a Gaussian trend, with an average value of 51.47 ± 20.34 nm. This increased polydispersity indicates the occurrence of secondary nucleation or partial coalescence events, where specific seeds grow more rapidly than others, resulting in significant size and contrast fluctuations among the particles.

For the 1:1 ratio (Figure 4e), the morphological diversity of the particles is significantly reduced, though the T-AuPs exhibit a substantially larger size. The corresponding histogram (Figure 4f) displays a trimodal distribution resulting from the convolution of three Gaussian functions centered at 103.98 ± 34.97 nm, 354.68 ± 60.65 nm and 674.78 ± 86.99 nm. The smallest polyhedral particles observed in this image correspond to unreacted seeds, whereas the largest triangular particles likely resulted from rapid growth or coalescence between neighboring particles. The histogram shown in Figure 4f was derived from over 300 particles, providing a statistically reliable representation of size distribution, and the trimodal distribution perfectly supports the “burst-growth” kinetic theory—some particles stay small (seeds), while others consume the precursor in a massive growth spurt. A summary of the mean particle sizes for all molar ratios is provided in Table 2.

**Table 2:** Mean Particle Size and Standard Deviations for the T-AuPs, Determined from the BSE-SEM Images Presented in Figure 4

HAuCl_4_/NaBH_4_	HAuCl_4_ (mL)	NaBH_4_ (mL)	diameter (nm)
1:12	0.025	0.3	31.3 ± 5.06
1:6	0.025	0.15	51.47 ± 20.34
1:1	0.3	0.3	103.98 ± 34.97; 354.68 ± 60.65; 674.78 ± 86.99

### 3.4 TEM Structural Characterization

To further investigate the internal structure, thickness, crystallinity, as well as structural features related to the growth mechanism of the AuPs, TEM analysis was performed (Figure 5).

**Figure 5:**
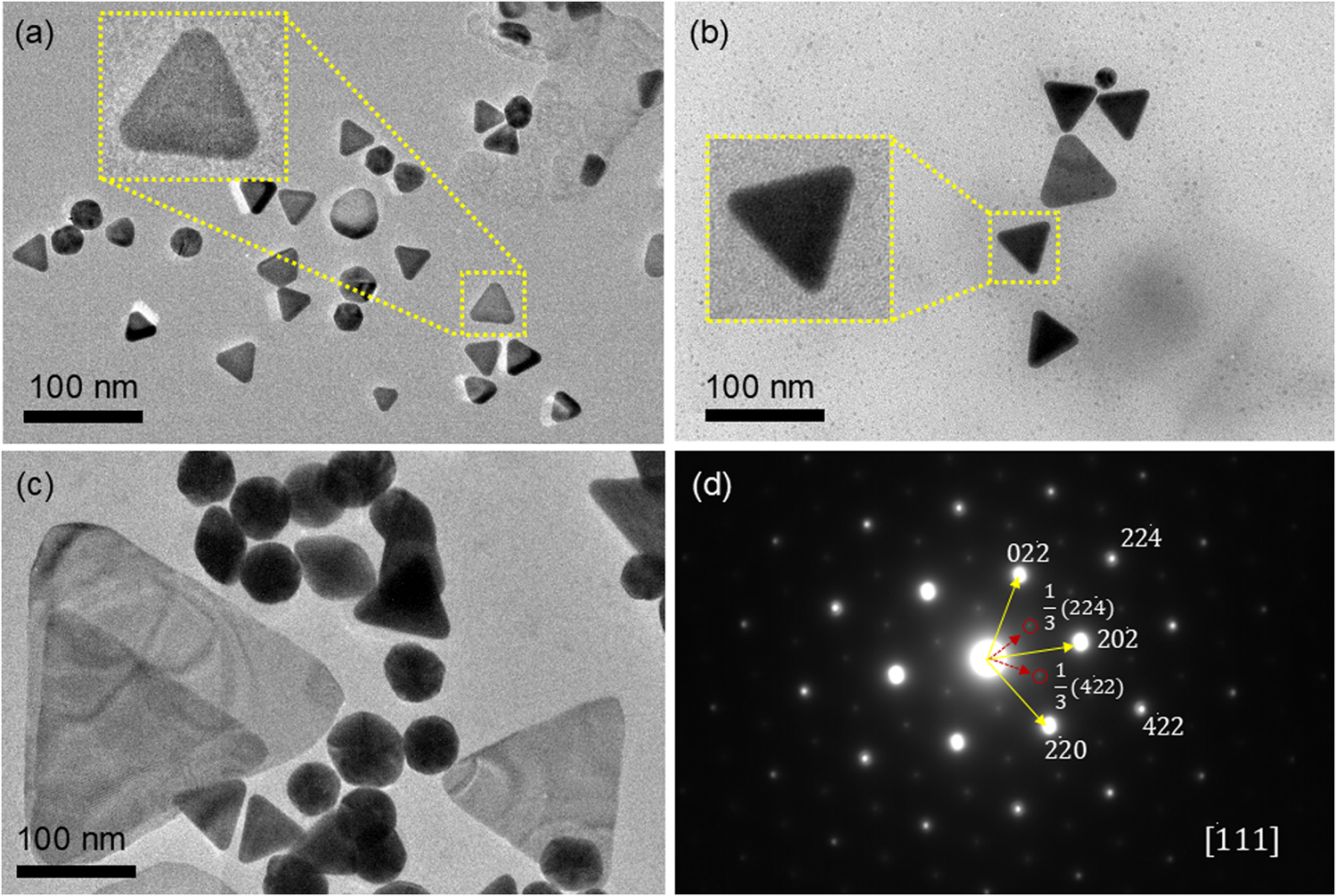
Bright field TEM images of the T-AuPs synthesized at various HAuCl_4_/NaBH_4_ molar ratios: (a) 1:12, (b) 1:6, and (c) 1:1. Note in (c) the existence of bend-contour fringes. (d) SAED pattern of an individual 320 nm T-AuPs from (c).

The 1:12 ratio (Figure 5a) exhibits uniform contrast, smooth surfaces, and rounded tips, which are indicative of controlled anisotropic growth. For the 1:6 ratio (Figure 5b), the darker image contrast suggests greater thickness compared to the 1:12 sample. In contrast, the 1:1 ratio (Figure 5c) shows larger size and rounded tips, yet reduced thickness, as evidenced by the observation of bend-contour fringes. These fringes appear when a thin crystal is slightly deformed or bent, which happens more easily in high-aspect-ratio plates (large but very thin) than in chunky particles. Therefore, the 1:1 ratio’s thickness is lower despite its larger lateral size is an important observation because it suggests that at the 1:1 ratio, the particles grow as thin plates rather than thick prisms.

Selected area electron diffraction (SAED) patterns confirmed the face-centered cubic (FCC) structure of individual T-AuPs particle oriented along the [1̅11] zone axis (Figure 5d). Notably, in addition to the primary FCC reflections, extra diffraction spots are observed in this SAED pattern, corresponding to an interplanar spacing of 0.2531 nm (highlighted in red). This value is slightly larger than the 0.2355 nm spacing characteristic of the {111} planes. These additional reflections are indexed as 1/3{422}, which is commonly attributed to surface reconstruction or periodic modulation effects typically observed in T-AuPs.^54–56^ The presence of the 1/3{422} reflections are “formally forbidden” in bulk FCC gold but appear in these nanostructures due to the presence of stacking faults or twin boundaries parallel to the {111} faces. They are a classic signature of thin, flat gold nanoprisms.^54–56^

### 3.5 SERS Performance of the Obtained Gold Particles

After establishing the relationship between reaction kinetics, plasmonic response, and morphology, the functional performance of the synthesized AuPs was evaluated using Surface-Enhanced Raman Scattering (SERS). Because SERS intensity depends strongly on electromagnetic field localization at particle edges and interparticle junctions (“hotspots”), this technique serves as a sensitive probe for the structure–property relationship differences generated under the various growth regimes. The triangular particles (especially those with sharp vertices from the 1:6 ratio) should theoretically provide much higher enhancement than the rounded spheres from the 1:12 ratio.

The Raman spectra are dominated by vibrational modes associated with CTAC molecules adsorbed on the gold surface, which act as intrinsic probe molecules without the need for additional analytes. By using the CTAC signal itself, we are essentially probing the chemical environment at the interface. This is a clever way to compare the “SERS activity” of the different shapes (spheres vs triangles) because the concentration of CTAC at the surface is relatively consistent across samples. The primary experimental vibrational modes and their corresponding assignments are summarized in Table 3. Characteristic CTAC vibrations, including CH_2_ scissoring,^57–60^ CH_3_ deformation,^58,61,62^ and C–C stretching,^57,59,60,63^ were clearly observed. These signals confirm the presence of the surfactant on the particle surface, which can effectively serve a model organic analyte.

**Table 3:** Experimental Raman Vibrational Modes and Assignments for the AuPs Synthesized at Various HAuCl_4_/NaBH_4_ Molar Ratios

Raman shift (intensity) ν_exp_ (cm^–1^)				
molar ratio (HAuCl_4_/NaBH_4_)				
1:12	1:6	1:1	Raman shift ν_exp_ (cm^–1^) [ref]	vibrational mode
1472 (363)	1471 (420)	1470 (4271)	∼1471 ^57–60^	**δ**_**sci**_(**CH**_2_) scissoring
1426 (479)	1431 (278)	1432 (2453)	∼1435 ^58,61,62^	**δ**_**sci**_(**CH**_2_) + **δ**_**def**_(**CH**_3_)
--	--	1404 (802)	∼1400 ^58^	**δ**_**wag**_(**CH**_2_) + **δ**_**def**_(**CH**_3_)
1356 (564)	1357 (154)	1359 (1069)	∼1349 ^61,64^	**δ**_**wag**_(**CH**_2_)
--	1331 (137)	1334 (1119)	∼1338 ^57,64,65^	**δ**_**tw**_(**CH**_2_)-twisting
1249 (1106)	1251 (1429)	1250 (10583)	∼1252 ^57–59^	**δ**_**wag**_(**CH**_2_)
1131 (630)	1131 (745)	1130 (5346)	∼1138 ^59,60,63^	**ν**(**C**–**C**)stretching
1059 (422)	1064 (320)	1060 (2261)	∼1060 ^60,63^	**ν**(**C**–**C**)stretching
985 (297)	--	988 (569)	∼988 ^57,60^	**ν**(**C**–**C**)stretching
--	956 (220)	955 (1119)	∼964 ^58,60^	**ρ**(**CH**_2_)rocking + **ν**(**C**–**N** ^+^) stretching
--	890 (212)	887 (1024)	∼891 ^57,58,65^	**δ**_**def**_(**CH**_3_)
292 (405)	291 (662)	296 (544)	∼294 ^65^	**δ**_**def**_(**C**_4_ **N** ^+^)

The SERS response varies markedly with the HAuCl_4_/NaBH_4_ molar ratio, as shown in Figure 6. The 1:6 and 1:12 samples (Figure 6a, 6b) exhibited progressively weaker signals, whereas the 1:1 sample (Figure 6c) displayed the most intense bands, with an intensity increase of over 9-fold compared to the weakest signal.

**Figure 6:**
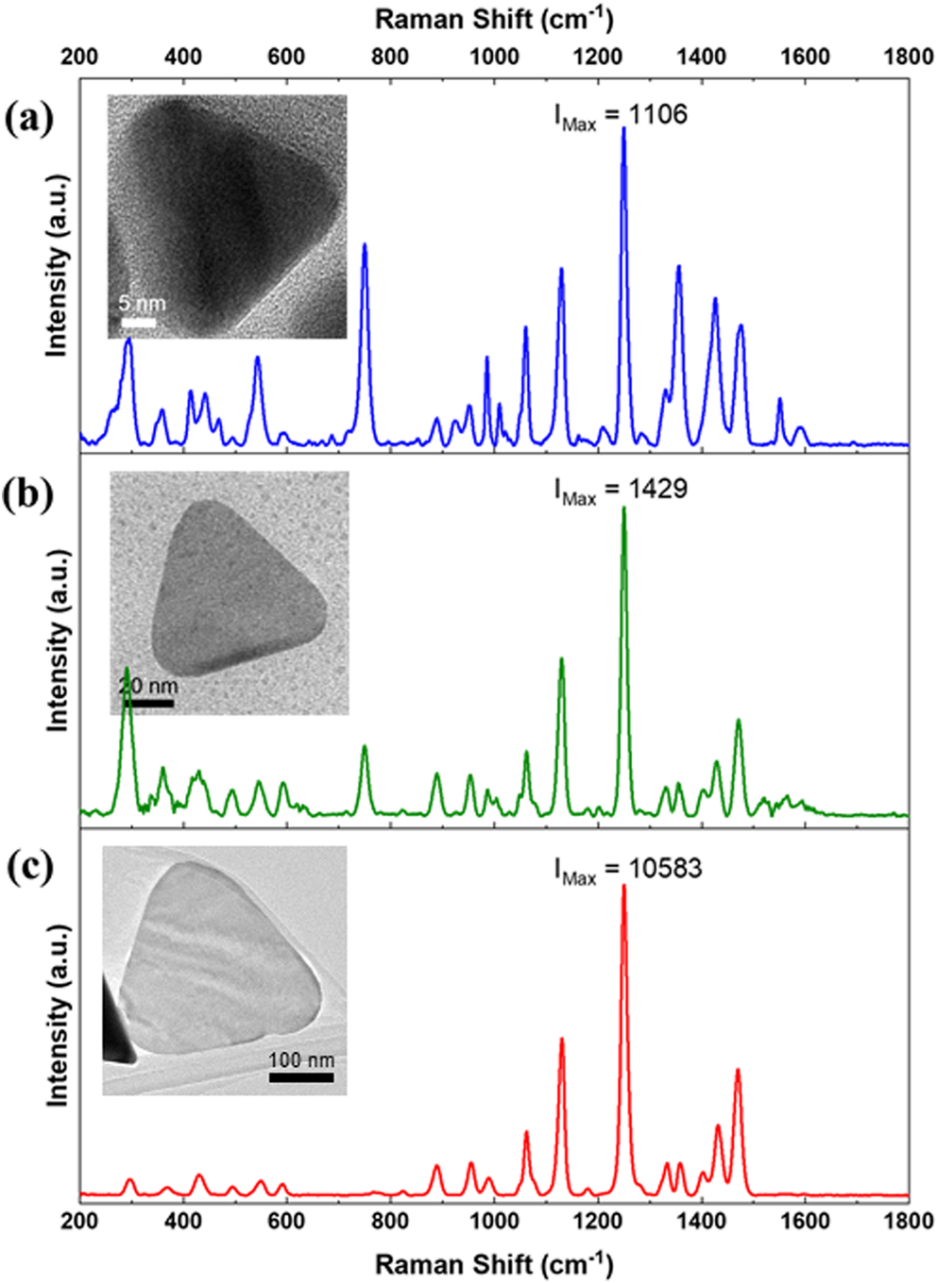
SERS activity of AuPs synthesized at various HAuCl_4_/NaBH_4_ molar ratios: (a) 1:12, (b) 1:6, and (c) 1:1. The samples were prepared with a constant volume of CTAC (630 μL of 25 wt % solution) dispersed in 5 mL of distilled water. The insets display the characteristic contrast of the T-AuPs observed in each respective ratio.

The differences in SERS intensity correlate directly with the nucleation and growth regimes identified previously. In the 1:1 system, slower nucleation followed by rapid burst growth surely produces larger triangular plates and regions of close particle proximity. These configurations promote strong plasmonic coupling and the formation of electromagnetic “hotspots”, particularly at sharp vertices and interparticle junctions, resulting in the highest near-field enhancement and Raman signal.

To further support the structure–performance relationship, a semiquantitative comparison of the SERS response was performed by normalizing the intensity of the **δ**_**wag**_(**CH**_2_) vibrational bands across all samples. The 1:1 system exhibited an enhancement of approximately 9-fold relative to the 1:12 system, consistent with the increased density of plasmonic hot spots. This behavior correlates with the optical response, where the broader and red-shifted LSPR band observed in the 1:1 sample indicates stronger interparticle coupling and enhanced local electromagnetic fields.

In contrast, the narrower plasmonic bands in the 1:6 and 1:12 systems reflect more isolated nanoparticles with reduced coupling efficiency. Additionally, the larger triangular nanoplates and their propensity to form junctions, as evidenced by SEM and TEM analysis, increase the probability of electromagnetic field confinement at edges and interparticle gaps. These results provide quantitative support for the direct relationship between nanoparticle morphology, plasmonic coupling, and SERS enhancement.

The fact that the 1:1 ratio provides the highest SERS signal despite its “complex: trimodal distribution suggests that the large triangular plates or the specific junctions between the “burst-growth” particles are creating exceptionally efficient electromagnetic “hotspots”. In contrast, the 1:6 and 1:12 systems experience faster nucleation, generating a higher density of smaller particles that remain more spatially isolated. The reduced electromagnetic coupling between these separated particles limits local field amplification and results in significantly weaker SERS signals.

To distinguish between intrinsic molecular-surface interactions and aggregation-induced electromagnetic effects, it is important to consider both the spectroscopic signatures and the morphological characteristics of the samples. The Raman spectra for all systems exhibit similar vibrational modes associated with CTAC, indicating that the chemical environment and adsorption behavior of the probe molecules remain largely unchanged across different synthesis conditions.

In contrast, the UV–Vis spectra of the 1:1 system displays a pronounced redshift and spectral broadening of the LSPR band, which are indicative of strong interparticle coupling and the formation of plasmonic hot spots. SEM and TEM analyses further confirm the presence of closely spaced nanoparticles and junction-like structures in this system. These observations suggest that the enhanced SERS response is predominantly governed by electromagnetic field amplification arising from nanoparticle aggregation and coupling effects, rather than from significant variations in intrinsic molecule–surface interactions. This distinction highlights the critical role of nanoscale spatial organization in determining SERS performance.

These results demonstrate that the precursor ratio dictates reaction kinetics, which in turn governs particle morphology and plasmonic coupling. Consequently, kinetic regulation provides a practical bottom-up route to tune the SERS performance of T-AuPs without the need for postsynthetic modification. This control enables their application as highly effective substrates for molecular detection and plasmonic sensing.

## 4 Conclusions

The fundamental link between precursor stoichiometry, reaction kinetics, structural evolution, and the resulting plasmonic performance of gold particles was successfully established. In this seeded-growth approach, the HAuCl_4_/NaBH_4_ molar ratio governs the kinetic balance between nucleation and growth. Specifically, high concentrations of NaBH4 (1:12 ratio) favor rapid nucleation and diffusion-limited growth, yielding smaller, uniform spheres and decahedrons. Conversely, a 1:1 ratio induces a chemically controlled “burst-growth” regime due to the stabilization of [AuCl_4_]^−^–CTA^+^ ion pairs, promoting the formation of large, thin triangular plates.

The optical response of these colloidal systems is highly sensitive to these kinetic shifts; the transition from a narrow, single LSPR band to a trimodal distribution in the 1:1 system reflects the coexistence of multiple growth pathways and increased particle size. The functional utility of these T-AuPs was validated through SERS, where the 1:1 system outperformed all other ratios. The larger plate dimensions and close interparticle proximity facilitate superior plasmonic coupling and the creation of electromagnetic ″hotspots″ at sharp vertices, resulting in a 6-fold increase in SERS sensitivity.

Overall, this work demonstrates that bottom-up kinetic engineering is a powerful, practical, and scalable strategy for designing high-performance plasmonic substrates. By simply adjusting the initial precursor ratios, the SERS performance can be precisely tuned without postsynthetic modification, providing a cost-effective pathway for developing advanced molecular sensors.

## Supplementary Material



## Notes

Theoretical background of the reaction pathway; UV–Vis calibration curves; and complete kinetic fitting results (PDF)
