# Supplementary material for: SERS Activity in Gold Particles Obtained via a Modified Seeded-Growth Method
Source: ACS Omega. 2026 Jun 9;11(24):36311–21. doi: 10.1021/acsomega.6c04232 (PMC13295057; doi:10.1021/acsomega.6c04232)
Supplement: Supplementary file 1 [file ao6c04232_si_001.pdf]

SUPPORTING INFORMATION

SERS Activity in Gold Particles obtained via a Modified Seeded-Growth Method

Jathziri Avalos-Grajales[1], Mario Alejandro Millán-Franco[1], Lucia Ortega-Cabello[2], Edgar Eduardo Mosquera-Vargas[3,4], José Reyes-Gasga[1]**

[1]Instituto de Física, Universidad Nacional Autónoma de México, 04510 Ciudad de México, México

[2]Departamento de Sistemas Biológicos, Universidad Autónoma Metropolitana, 04960 Ciudad de México, México

[3]Grupo de Transiciones de Fase y Materiales Funcionales (GTFMF), Departamento de Física, Universidad del Valle, Santiago de Cali, Colombia

[4]Centro de Excelencia en Nuevos Materiales (CENM), Universidad del Valle, Santiago de Cali, Colombia

THEORETICAL BACKGROUND

Reaction Pathways in Gold Nanotriangles Synthesis. The synthesis of T-AuPs using a seed-mediated growth approach involves a sequence of chemical reactions that reduce $HAuCl_4$ and led to the formation of T-AuPs with controlled morphology. CTAC plays a key role as both stabilizing and shape-directing agent, promoting the formation of triangular particles.

The process begins with the formation of a CTA-stabilized $AuCl_4^-$ complex, generated by mixing $HAuCl_4$ with CTAC. The solution turns bright yellow, indicating the successful formation of the complex between the tetrachloroaurate ion ($AuCl_4^-$) and the cationic surfactant CTAC [1]. This step is crucial, as it stabilizes the $AuCl_4^-$ ions and prepares them for subsequent reduction:

$$\mathrm{HAuCl}_{4(aq)} + \mathrm{CTAC}_{(aq)} \longrightarrow \mathrm{AuCl}_4 - \mathrm{CTA}_{(aq)} + \mathrm{H}^+_{(g)} \quad (1)$$

This complex is subsequently reduced using $NaBH_4$, yielding small gold seeds. This reduction step is critical for the nucleation and establishes the foundation for subsequent particle growth.

$$2\mathrm{NaBH}_{4(aq)} + \mathrm{AuCl}_4 - \mathrm{CTA}_{(aq)} \longrightarrow \mathrm{AuSeeds(CTA)}_{(s)} + 2\mathrm{NaCl}_{(aq)} + \mathrm{B}_2\mathrm{H}_{6(g)} + 2\mathrm{HCl}_{(aq)} \quad (2)$$

This equation highlights the transformation of Au^{3+} ions into metallic gold (Au^0), stabilized by CTAC, while accounting for the byproducts formed during the reaction. The resulting seeds act as nucleation centers for anisotropic growth. In the presence of a milder reducing agent, AA, additional gold ions from $HAuCl_4$ are reduced and deposited onto the seeds:

$$\mathrm{AA}_{(aq)} + \mathrm{AuCl}_4 - \mathrm{CTA}_{(aq)} + \mathrm{H}^+ \longrightarrow \mathrm{AuSeeds(CTA)}_{(s)} + 4\mathrm{HCl}_{(aq)} + \mathrm{AA\ oxidado}_{(aq)} \quad (3)$$

$$AuSeeds(CTA)_{(s)} + HAuCl_{4(aq)} + NaI_{(aq)} \longrightarrow AuPs_{(s)} + NaCl_{(aq)} + I^{-}_{(aq)} + CTA^{+}_{(aq)}$$
(4)

Due to gas evolution, a rest period of approximately 2 hours is typically required to ensure the complete decomposition of residual $NaBH_4$ and to promote seed stabilization [2].

Chemical Kinetics. The study of particle nucleation and growth is commonly approached using kinetic models that describe concentration changes and elucidate the underlying reaction mechanisms.

As reactant concentrations change, the reaction rate also varies. This dependence is studied experimentally by measuring how the rate responds to different initial reactant concentrations, allowing the establishment of a proportional relationship between the rate and the concentration of one or more species. This relationship is known as the rate law (or rate equation), and the proportionality constant is referred to as the rate constant (k) [3, 4]. The general form of the rate law is:

$$Reaction\ rate = \frac{-dA}{dt} = k[A]^m[B]^n \quad (5)$$

The exponents in the rate law (reaction orders) do not necessarily correspond to the stoichiometric coefficients and must be determined experimentally [5].

The reaction rate is analyzed at constant initial reactant concentrations to determine the reaction order. The "initial rate method" measures the rate immediately after mixing, thereby reflecting the starting concentrations. This rate is obtained graphically from the early portion of the experimental data [6–8].

A first-order reaction is characterized by a rate law in which the sum of the exponents equals one. In such cases, the rate is directly proportional to the concentration of a single reactant. Although other reactants may be present in the system, their concentrations remain nearly constant during the reaction and therefore have little influence on the rate. The rate law for a first-order reaction is [9, 10]:

$$Reaction\ rate = \frac{-dA}{dt} = k[A] \quad (6)$$

On the other hand, a second-order reaction is characterized by a rate law in which the sum of exponents equals two. This implies that the rate is proportional either to the square of the concentration of a single reactant or to the product of the concentration of two reactants. The corresponding rate laws are: [10, 11]

$$Reaction\ rate = \frac{-dA}{dt} = k[A]^2 \quad (7)$$

$$Reaction\ rate = \frac{-dA}{dt} = k[A][B] \quad (8)$$

In our study, the analysis was simplified using pseudo-first- or pseudo-second-order kinetic models, in which the rate depends linearly on the $HAuCl_4$ concentration, while the effects of other reactants are incorporated into the apparent rate constant (k'). To monitor reactant concentrations during the reaction, we applied the Beer-Lambert law, which relates absorbance to concentration [12, 13]. It is expressed as:

$$A = \varepsilon c l \quad (10)$$

where A is the absorbance, ε is the molar absorptivity (extinction coefficient), c is the concentration of the absorbing species, and l is the optical path length through the solution. This law is fundamental to our study, as it enables the determination of the concentrations of species such as $HAuCl_4$, T-AuPs, and reaction intermediates during the synthesis process by monitoring changes in absorbance over time.

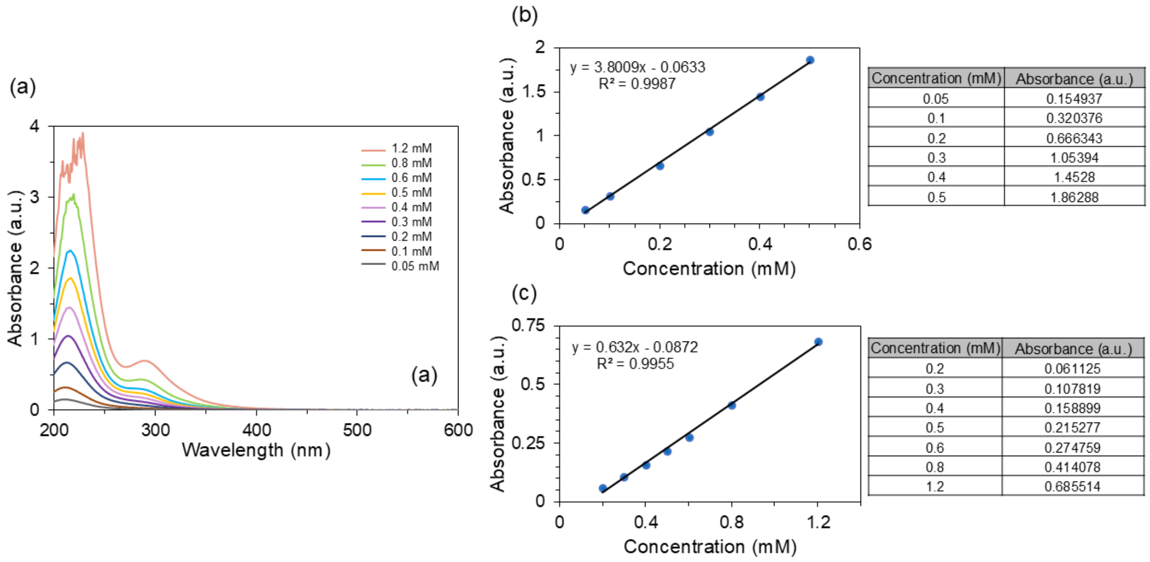

Concentration (mM)	Absorbance (a.u.)
0.05	0.154937
0.1	0.320376
0.2	0.666343
0.3	1.05394
0.4	1.4528
0.5	1.86288

Concentration (mM)	Absorbance (a.u.)
0.2	0.061125
0.3	0.107819
0.4	0.158899
0.5	0.215277
0.6	0.274759
0.8	0.414078
1.2	0.685514

Figure S1. UV-Vis spectroscopic monitoring and calibration of $HAuCl_4$ in aqueous solution. a) UV-Vis spectra of $HAuCl_4$ solutions at varying concentrations; the two distinct peaks correspond to electronic transitions of the $[AuCl_4]^-$ species. b) Primary calibration curve derived from the main absorption band at 220 nm. c) Secondary calibration curve derived from the second absorption maximum at 294 nm, including the linear fit and associated data parameter table.

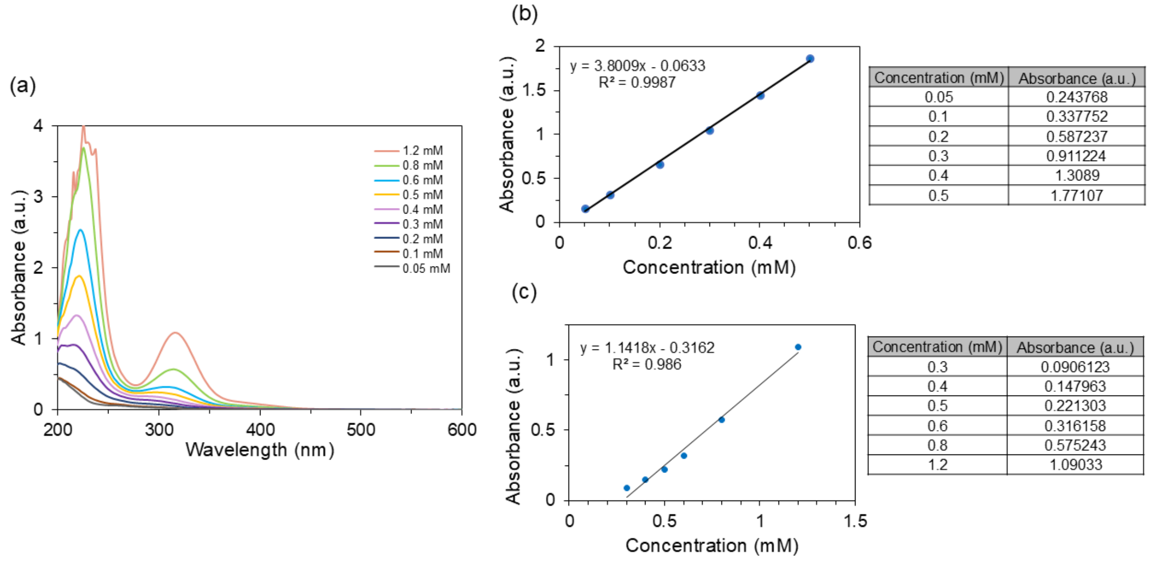

Concentration (mM)	Absorbance (a.u.)
0.05	0.243768
0.1	0.337752
0.2	0.587237
0.3	0.911224
0.4	1.3089
0.5	1.77107

Concentration (mM)	Absorbance (a.u.)
0.3	0.0906123
0.4	0.147963
0.5	0.221303
0.6	0.316158
0.8	0.575243
1.2	1.09033

Figure S2. Spectroscopic analysis of $HAuCl_4$ in the presence of CTAC. a) UV-Vis spectra of $HAuCl_4$ solutions with a constant CTAC concentration; the spectral shifts indicate the formation of the $[AuCl_4]^-$ –CTA^+ ion-pair complex. b-c) Calibration curves at 220 and 320 nm corresponding to transitions influenced by surfactant–precursor interactions. Data points were fitted to linear models; the associated absorbance values and fitting parameters are provided in the inset tables.

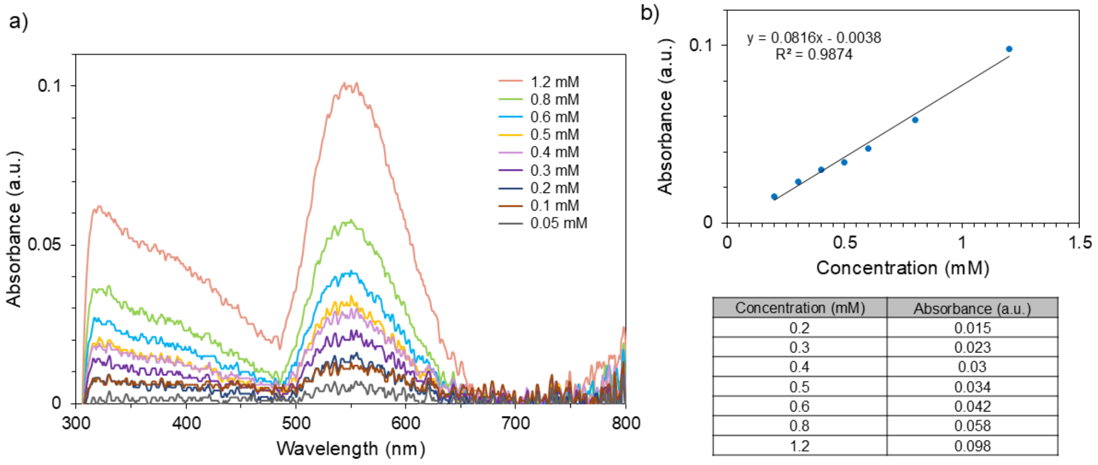

Concentration (mM)	Absorbance (a.u.)
0.2	0.015
0.3	0.023
0.4	0.03
0.5	0.034
0.6	0.042
0.8	0.058
1.2	0.098

Figure S3. UV-Vis response and calibration of $HAuCl_4$ in presence of NaI and ascorbic acid (AA). a) UV-Vis spectra of $HAuCl_4$ solutions treated with NaI and AA under in aqueous media. b) Calibration curve derived from the absorbance at 550 nm, including the linear fit and the associated data table.

Table S1. Summary of the kinetic fitting parameters for pseudo–first order and pseudo–second-order models at various $HAuCl_4$:$NaBH_4$ molar ratios, including both statistically significant fits and instances where data did not adhere to the models.

Moral ratio ($HAuCl_4$:$NaBH_4$)	R^2	k'	Stage of the synthesis	Evaluated model
1:1	0.9098	-0.0003 ± 1.42 $x10^{-4}$ s^{-1}	Nucleation	Pseudo-first order
	0.9124	0.0001 ± 0.16 $x10^{-4}$ mol s^{-1}	Nucleation	Pseudo-second order
	0.9062	0.038 ± 0.0055 s^{-1}	Growth	Pseudo-first order
	0.7364	-14.05 ± 3.76 mol s^{-1}	Growth	Pseudo-second order
1:6	0.9176	0.002 ± 2.54 $x10^{-4}$ s^{-1}	Nucleation	Pseudo-first order

	0.8964	-0.007 ± 2.54 x10^{-4} mol s^{-1}	Nucleation	Pseudo-second order
	0.9087	0.0027 ± 3.29 x10^{-4} s^{-1}	Growth	Pseudo-first order
	0.8886	-0.0025 ± 3.34 x10^{-4} mol s^{-1}	Growth	Pseudo-second order
**1:12**	0.8978	-0.0008 ± 1.12 x10^{-4} s^{-1}	Nucleation	Pseudo-first order
	0.9074	0.0039 ± 4.73 x10^{-4} mol s^{-1}	Nucleation	Pseudo-second order
	0.9002	0.0028 ± 3.66 x10^{-4} s^{-1}	Growth	Pseudo-first order
	0.885	-0.282 ± 0.040 mol s^{-1}	Growth	Pseudo-second order

REFERENCES

1. Khan Z, Singh T, Hussain JI, Hashmi AA (2013) Au(III)–CTAB reduction by ascorbic acid: Preparation and characterization of gold nanoparticles. Colloids Surf B Biointerfaces 104:11–17. https://doi.org/10.1016/j.colsurfb.2012.11.017

2. Podlesnaia E, Csáki A, Fritzsche W (2021) Time Optimization of Seed-Mediated Gold Nanotriangle Synthesis Based on Kinetic Studies. Nanomaterials 11:1049. https://doi.org/10.3390/nano11041049

3. Cortright RD, Dumesic JA (2001) Kinetics of heterogeneous catalytic reactions: Analysis of reaction schemes. pp 161–264

4. Laidler KJ (2013) Reaction kinetics: homogeneous gas reactions

5. Cortright RD, Dumesic JA (2001) Kinetics of heterogeneous catalytic reactions: Analysis of reaction schemes. pp 161–264

6. Koerner H, MacCuspie RI, Park K, Vaia RA (2012) In Situ UV/Vis, SAXS, and TEM Study of Single-Phase Gold Nanoparticle Growth. Chemistry of Materials 24:981–995. https://doi.org/10.1021/cm202633v

7. Vallance C (2017) An Introduction to Chemical Kinetics. Morgan & Claypool Publishers

8. Dai Z, Flatberg G, A. Preisig H, Deng L (2018) Kinetic Studies of Fenton Oxidation Reaction by UV-VIS Spectroscopy. J Lab Chem Educ 6:141–147. https://doi.org/10.5923/j.jlce.20180605.01

9. Gaffney JS, Marley NA (2018) General Chemistry for Engineers. Elsevier

10. Levenspiel O (1999) Chemical Reaction Engineering, 3rd Edition. New York

11. Donaldson DJ, Wren SN (2015) CHEMISTRY OF THE ATMOSPHERE | Laboratory Kinetics. In: Encyclopedia of Atmospheric Sciences. Elsevier, pp 356–362

12. Mäntele W, Deniz E (2017) UV–VIS absorption spectroscopy: Lambert-Beer reloaded. Spectrochim Acta A Mol Biomol Spectrosc 173:965–968. https://doi.org/10.1016/j.saa.2016.09.037

13. Mosorov V (2017) The Lambert-Beer law in time domain form and its application. Applied Radiation and Isotopes 128:1–5. https://doi.org/10.1016/j.apradiso.2017.06.039
